# Insights into the Roles of the Sideroflexins/SLC56 Family in Iron Homeostasis and Iron-Sulfur Biogenesis

**DOI:** 10.3390/biomedicines9020103

**Published:** 2021-01-21

**Authors:** Nesrine Tifoun, José M. De las Heras, Arnaud Guillaume, Sylvina Bouleau, Bernard Mignotte, Nathalie Le Floch

**Affiliations:** 1 LGBC, UVSQ, Université Paris-Saclay, 78000 Versailles, France; nesrine.tifoun2@uvsq.fr (N.T.); jos.de-las-heras-chanes@uvsq.fr (J.M.D.l.H.); arnaud.guillaume@uvsq.fr (A.G.); sylvina.bouleau@uvsq.fr (S.B.); bernard.mignotte@uvsq.fr (B.M.); 2 École Pratique des Hautes Études, PSL University, 75014 Paris, France; 3 GCGP Department, IUT de Vélizy/Rambouillet, UVSQ, Université Paris-Saclay, 78120 Rambouillet, France

**Keywords:** sideroflexin, mitochondria, mitochondrial transporters, iron homeostasis, iron-sulfur cluster, heme biosynthesis, one-carbon metabolism, ferroptosis, ferritinophagy

## Abstract

Sideroflexins (SLC56 family) are highly conserved multi-spanning transmembrane proteins inserted in the inner mitochondrial membrane in eukaryotes. Few data are available on their molecular function, but since their first description, they were thought to be metabolite transporters probably required for iron utilization inside the mitochondrion. Such as numerous mitochondrial transporters, sideroflexins remain poorly characterized. The prototypic member SFXN1 has been recently identified as the previously unknown mitochondrial transporter of serine. Nevertheless, pending questions on the molecular function of sideroflexins remain unsolved, especially their link with iron metabolism. Here, we review the current knowledge on sideroflexins, their presumed mitochondrial functions and the sparse—but growing—evidence linking sideroflexins to iron homeostasis and iron-sulfur cluster biogenesis. Since an imbalance in iron homeostasis can be detrimental at the cellular and organismal levels, we also investigate the relationship between sideroflexins, iron and physiological disorders. Investigating Sideroflexins’ functions constitutes an emerging research field of great interest and will certainly lead to the main discoveries of mitochondrial physio-pathology.

## 1. Sideroflexins: From Structure to Function

### 1.1. Sideroflexins from an Historical Point of View

The mitochondrion is at the crossroad of key metabolic pathways (energy metabolism, central carbon metabolism, one carbon metabolism, lipid, nucleotides and amino acids synthesis, etc.) and is a key player in cell fate and response to stress or infection. In order to ensure its essential functions within the cell, the mitochondrion requires a wide variety of enzymes and transporters. Among these proteins, sideroflexins (SFXN) form a family of recently discovered mitochondrial proteins whose cell functions are progressively being specified. The first mention of the name “sideroflexin” appeared in 2001 [1]. Since then, a few studies have been dedicated to SFXN proteins, and at the time we were writing this review, only 24 articles were retrieved in Pubmed using the keyword “sideroflexin”. Pioneers in the SFXN field, Fleming et al. identified a mutation affecting the *Sfxn1* gene in the flexed-tail mouse and proposed that the loss of Sfxn1 was responsible for the sideroblastic anemia phenotype. Thus, SFXN owe their name to the mice in which they were discovered (SIDEROblastic anemia and FLEXed-tail mouse) [1]. However, it should be noticed that the causal link between the mutation in the *Sfxn1* gene and the phenotype of flexed-tail mice has not been clearly established yet. It was even questioned following a study showing that flexed tailed mice also had a mutation of the *Madh5/Smad5* gene, involved in the BMP pathway, which could explain the anemia and *flexed-tail* phenotype [2,3].

### 1.2. The Sideroflexin Family: From Genes to Proteins

Sideroflexins (forming the SFXN/SLC56 family of mitochondrial transporters [4]) are highly conserved throughout eukaryotes. Only one sideroflexin is found in yeast (Fsf1 for Fungal sideroflexin 1), whereas there are two SFXNs in *Drosophila* (dSfxn1/3 and dSfxn2) and five SFXN (SFXN1-5) in vertebrates [1,5,6,7]. Our purpose is not to give an extensive overview of SFXN tissue distribution in this review, but some data are available in the literature. For example, SFXN1 mRNA levels in normal tissues and human cancers, as well as tissue distribution of the five human SFXN, are available in Reference [8].

**Figure 1:**
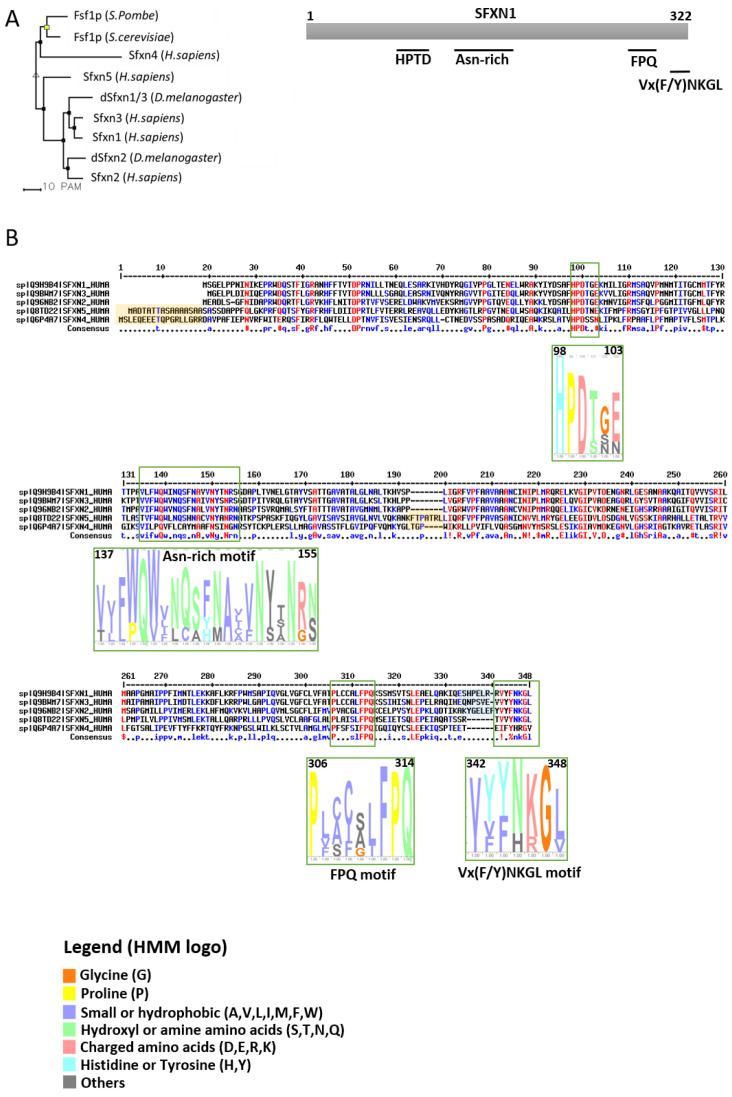
Sideroflexins (SFXNs) form a family of conserved proteins in *Eukarya*. (**A**). Left panel: Phylogenetic tree obtained using the MultiAlin software (http://multalin.toulouse.inra.fr/multalin/) [10]. Right panel: scheme of the SFXN1 protein and its conserved motifs. (**B**). Alignment of human SFXNs protein sequences. Red amino acids are for high consensus levels (90%), the blue ones are for low consensus levels (50%). Meaning of symbols found in the consensus line: “!” is for Ile or Val, “$” is for Leu or Met, “%” is for Phe or Tyr,” #” is anyone of Asn, Asp, Glu, Gln. Conserved motifs are shown and highlighted using an HMM logo created using Skyline (http://skylign.org/) with consensus colors for amino acids according to the ClustalX coloring scheme.

SFXNs homologues display a high amino acid identity rate in mouse [1], xenopus [5] and human [8]. In humans, SFXN1 and SFXN3 share 76.56% identical amino acids, whereas there is 56.05% identity between SFXN1 and SFXN2 and only 22.04% between SFXN1 and SFXN4. An alignment of human SFXNs is shown in Figure 1. Identity rates between the different human, *Drosophila* and yeast sideroflexins proteins are described elsewhere [8,9]. The high degree of homology between SFXNs, especially between SFXN1 and SFXN3 in humans, suggests that sideroflexins ensure redundant functions, as it was proposed for the mitochondrial import of serine that seems to be mediated by SFXN1 [8]. This function will be detailed in the section dedicated to the role of SFXN in regulating mitochondrial metabolism (see Section 3.1). Among the five mammalian SFXNs, SFXN4 is the most divergent member, suggesting that this member does not share the same functions (Figure 1). Indeed, SFXN4 was not able to suppress defects caused by the concomitant loss of SFXN1 and SFXN3 in mammalian cells [8]. Interestingly, till now no study has been done to specifically uncover the Fsf1 function. Due to the high degree of similarity between fungal sideroflexin and SFXN proteins from higher eukaryotes, we think that studies on the functions of Fsf1 will certainly lead to huge advances in the SFXN field and may reveal a general function for this family of proteins.

### 1.3. Sideroflexins Are Mitochondrial Transporters Implicated in One-Carbon Metabolism

SFXNs possess four to six predicted transmembrane domains composed by α-helices revealed by in silico modeling [1,6,7]. These proteins share several highly conserved motifs, including a HPDT motif and an asparagine-rich sequence (Figure 1) [1,6]. The functions of those conserved motifs have not been uncovered yet. Recently, Gyimesi and Hediger performed an in silico analysis of human SFXN1-5 sequences and described six well-conserved regions that could be important for SFXNs activity [11]. Whether these conserved regions are essential for metabolite transport need to be further confirmed at the bench.

**Figure 2:**
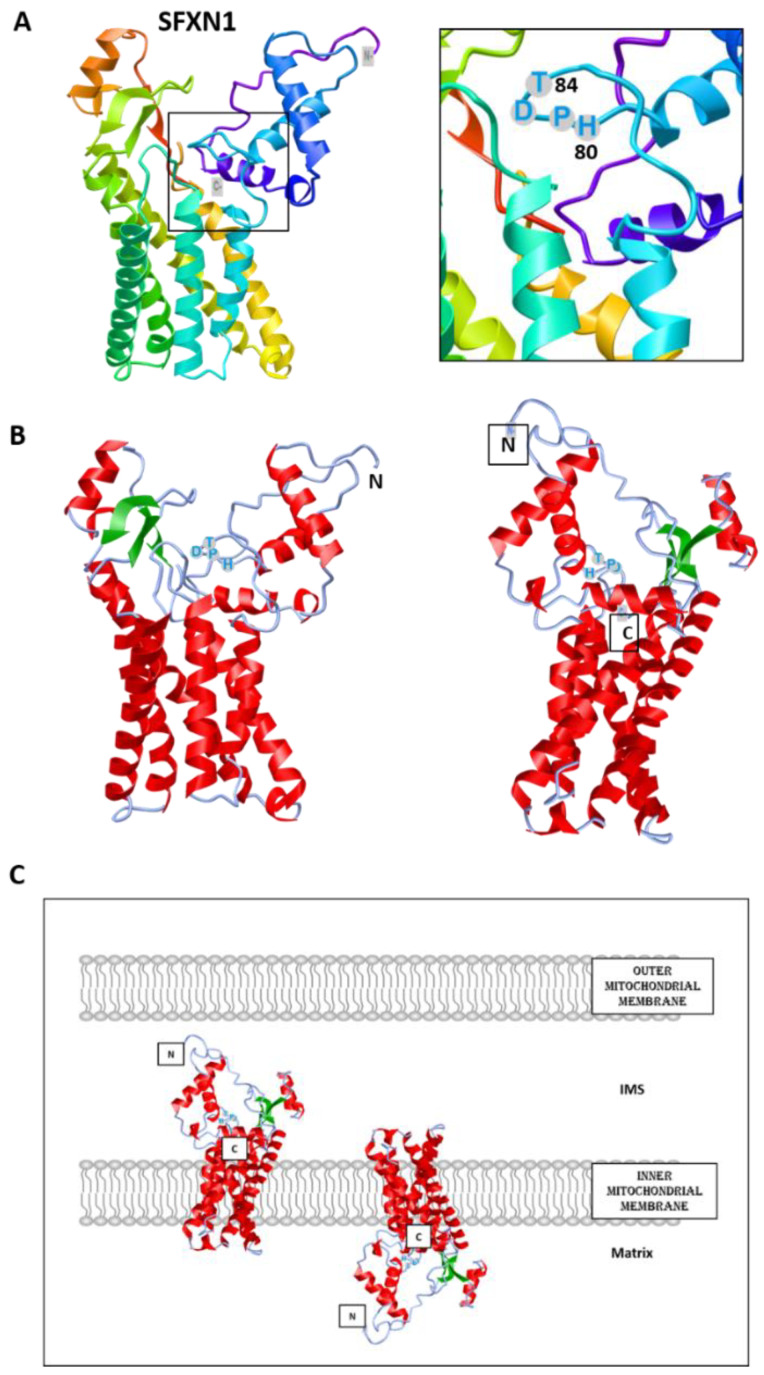
Predicted structure of human SFXN1. Structure prediction was obtained using trRosetta. The confidence of the predicted model shown here is very high (with estimated TM-score = 0.806). The model was built by trRosetta based on de novo folding, guided by deep learning restraints. iCn3D was used for the visualization of 3D structure [13]. (**A**). SFXN1 predicted structure reveals several alpha helices and beta strands. N and C termini are labelled. The inlet shows the position of the HPDT motif (aa 80–83), located just after the fourth helix. (**B**). Two views highlighting secondary structures (helices in red, beta sheets in green). (**C**). Models for SFXN1 insertion in the inner mitochondrial membrane.

To date, no crystal structure has been released for SFXNs. We thus tried to model SFXN tridimensional structure using the trRosetta software [12]. The SFXN1 predicted structure is shown in Figure 2. Interestingly, this structure reveals six internal alpha helices that may correspond to the transmembrane domain of SFXN1.

SFXN1 topology was recently investigated by APEX and classical biochemical experiments [14,15,16]. Acoba et al. [16] performed detergent extraction and protease-protection assays on HEK human cells and confirmed that endogenous SFXN1 is a mitochondrial protein inserted in the inner mitochondrial membrane (IMM). Furthermore, evidence was given for the presence of the N-terminus in the intermembrane space (IMS), but not in the matrix, contrary to what is predicted by a in silico analysis using Protter. According to biochemical data, the C-terminus seems to protrude in the matrix, in agreement with the previously proposed five transmembrane domains. However, our model is rather in agreement with a TM domain composed of six alpha helices, and if this predicted structure is correct, the N and C termini could be in the same mitochondrial compartment (Figure 2). The CryoEM structure of SFXN1 is thus needed to precise the three-dimensional structure of this carrier. Moreover, two recent studies investigated the mechanisms of SFXN1 mitochondrial import and shed light on the role of TIM22 and AGK2 in this process [16,17]. Evidence for a mitochondrial localization of SFXN is listed in Table 1.

Due to their predicted structure, showing several hydrophobic alpha helices, and their mitochondrial location, sideroflexins were proposed to be mitochondrial metabolite transporters. Rat Sfxn3 was presumed to be a tricarboxylate carrier (TCC), and later, Sfxn5 (also known as BBG-TCC) was reported to transport citrate in vitro [21,22]. However, it was only recently that a function of mitochondrial serine transporter was reported for SFXN1 [8].

By a bioinformatic analysis, the *S. cerevisiae* Fsf1 (YOR271cp) was proposed to be a candidate alpha-isopropylmalate transporter but no experimental data ascertained this function [23]. Similarly, the predicted Fsf1 protein from *Schizosaccharomyces pombe*, Spac17g6.15c, is annotated as a serine transporter in the database Pombase (https://www.pombase.org/) based on its homology with human SFXN1 [24,25], although it has not been extensively studied.

Since mice lacking Sfxn1 present similar features to that observed in human syndromes caused by a lack of pyridoxine or ALAS2 mutation (X-linked sideroblastic anemia), it was also proposed that Sfxn1 transports pyridoxine (B6 vitamin) inside the mitochondria [1,26]. Since pyridoxine is the precursor of pyridoxal phosphate that serves as a cofactor for ALAS2 (the erythroid specific enzyme catalyzing the first step of heme biosynthesis), SFXN1 could thus directly regulate heme biosynthesis. However, it has been recently reported that human SFXN1 is not able to transport pyridoxine in vitro [8]. Even if we cannot exclude that SFXN1 functions in a complex that is not fully reconstituted in in vitro assays, SFXN1 may not be the carrier for pyridoxine. Mtm1p, SLC25A39 yeast homologue, was suggested to import pyridoxal 5′-phosphate inside the mitochondria [27,28]. However, the substrate specificity of the SLC25A39 carrier remains unknown [29].

Thus, the main role of SFXN1 seems to be the mitochondrial serine import. Inside the mitochondrion, serine can be catabolized by the serine hydroxymethyl transferase (SHMT2) into glycine, an amino acid necessary for ALA synthesis (see Section 3.1). Thus, the lack of SFXN1 would lead to decreased mitochondrial levels of serine and glycine, leading to ALA synthesis impairment (see Section 4).

### 1.4. Sideroflexins in Disease

Hildick-Smith et al. described, for the first time, a human syndrome (combined oxidative phosphorylation deficiency-18, OMIM entry # 615578), which was directly associated with the lack of a member of the SFXN family, namely SFXN4 [19]. Patients showed macrocytic anemia and mitochondriopathy non-explainable by other causes, but the lack of SFXN4. Recently, a third patient with SFXN4 mutations was described by Sofou et al. [30]. The three patients with SFXN4 mutations presented with intrauterine growth retardation, mild to severe intellectual disabilities, microcephaly, neonatal lactic acidosis, macrocytic anemia and severe visual impairment. Sofou et al. reported optic nerve hypoplasia in the third case. More recently, some of the mechanisms that could explain those effects in humans were reported in the K562 erythroleukemic cell line [31]. Interestingly, SFXN4 loss-of-function leads to a general decrease in the levels of the respiratory chain complexes I-IV, which could be explained by an impaired Fe-S cluster synthesis, as evidenced by a Fe-S fluorescence assay (FeSFA). Nevertheless, Sofou et al. showed that the effect of SFXN4 decrease would be exclusively in Complex I, but not in the rest of the respiratory chain complexes after muscle biopsy [30]. Despite these discrepancies, which could be due to the different nature of the mutations analyzed in each case, it seems clear that Complex I activity is affected in both studies, which reinforces the hypothesis that SFXN4 could have a role, either direct or indirect, on Fe-S biosynthesis.

Besides the description of mutations in the *SFXN4* human gene causing the COXPD18 syndrome, *SFXN4* was also reported to be a predisposition gene for familial colorectal cancer (CRC). Hence, rare *SFXN4* truncating variants were identified in 3/96 CRC familial cases [32]. An aberrant expression of *SFXN1* and *SFXN5* was also reported in patients with breast cancer or gliomas [33,34].

## 2. Sideroflexins and Mitochondrial Respiration

### 2.1. Overview of the Mitochondrial Respiratory Complexes and the Place of Iron in RC

**Figure 3:**
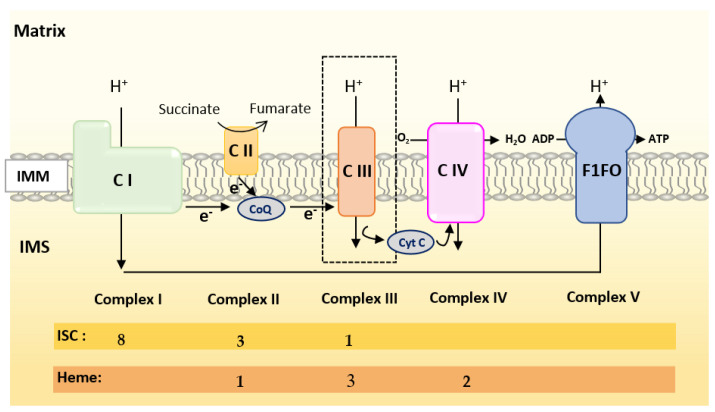
Scheme of the mitochondrial respiratory chain. For each complex, Iron Sulfur Cluster (ISC) and heme numbers are given.

Oxidative Phosphorylation (OXPHOS) couples the transport of electrons (through a series of mitochondrial respiratory complexes containing redox-active prosthetic groups) to the production of ATP by the mitochondrial ATP synthase, commonly referred to as the complex V of the respiratory chain (Figure 3). Respiratory complexes (RC) are arranged in supercomplexes (SC) and megacomplexes in the inner mitochondrial membrane [35,36]. The Electron Transport Chain (ETC) comprises four RCs (Complex I-IV) containing more than 70 nuclear DNA encoded subunits and 13 mitochondrial DNA (mtDNA) encoded subunits, some of which include iron-sulfur clusters (ISCs) or heme; those iron-containing groups are essential cofactors for electron transport from one complex to another [37,38]. The purpose of this review is not to give an extensive overview of the abundant literature on RC, so we invite the reader to refer to recent reviews for details on the composition, structure and biogenesis of RC [35,38,39].

Mammalian Complex I (NADH: Ubiquinone Oxireductase) is a L-shaped megastructure of about 1 MDa comprising 14 core subunits and up to 45 subunits. Among them, five essential subunits (NDUFV1, NUDFV2, NDUFS1, NDUFS7 and NDUFS8) bare the eight ISCs of CI (two [2Fe-2S] and six [4Fe-4S] clusters).

Mammalian Complex II, the smallest of the RC, is composed of only four subunits: succinate dehydrogenase [ubiquinone] flavoprotein (also known as Flavoprotein subunit of complex II, Fp, SDHA), succinate dehydrogenase [ubiquinone] iron-sulfur subunit (a Fe-S protein also named Ip or SDHB), the membrane-anchoring succinate dehydrogenase cytochrome b560 subunit (CybL, SDHC), and finally the succinate dehydrogenase (ubiquinone) cytochrome b small subunit (CybS, SDHD). These subunits are, respectively, encoded by the *SDHA*, *SDHB*, *SDHC* and *SDHD* nuclear genes. Fp/SDHA and Ip/SDHB are anchored to the IMM thanks to CybL/SDHC and CyBS/SDHD that are the membrane-anchoring subunits of CII. Complex II contains three ICSs ([2Fe-2S], [4Fe-4S] and [3Fe-4S] in SDHB) and a heme shared by SDHC and SDHD.

Mammalian Complex III (also known as bc1 complex) is a dimer made of monomers containing 11 subunits among which three are essential redox subunits: cytochrome b, cytochrome c1 and the Fe-S protein Cytochrome b-c1 complex subunit Rieske (Rieske, ISP, RISP, Rip1 are alternative names that can be found in the literature for this protein). Altogether, these catalytic subunits possess two heme b (Cyt b), a c-type heme (Cyt c1) and a [2Fe-2S] cluster (Rieske) [40]. Heme b is synthesized by Ferrochelatase (FECH), but the mechanism of its insertion into cytochrome b has not been fully elucidated [40].

Mammalian Complex IV contains three mitochondrially-encoded subunits (Cytochrome c oxidase subunit 1, 2 and 3) plus eleven subunits encoded by the nuclear genome. CIV possesses four redox-active metal centers, including heme a and heme a3, but no ISCs.

To summarize, Complex I is made of numerous subunits including 8 ISC-containing subunits but none containing heme. Complex IV presents four redox-active centers containing heme, but no ISC. Both Complexes II and III have ISC and heme containing subunits.

### 2.2. Current Knowledge on the Regulation of Mitochondrial Respiration by SFXN Proteins

Kory et al. reported decreased basal respiration in *SFXN1/SFXN3* double knockout Jurkat cells [8]. Whereas SFXN1 loss alone is not detrimental for respiration of intact cells [8,16], Acoba et al. reported a significant decrease in Oxygen Consumption Rates (OCR) of isolated mitochondria from HEK *SFXN1 KO* cells with CI, CII and CIII substrates (pyruvate, Glu, Gln, dimethyl-α-ketoglutarate, succinate and glycerol-3-phosphate) [16]. In human embryonic cells, the loss of SFXN1 leads to a marked decrease in the protein levels of three subunits of the Complex III and to a lesser extent in Complex II subunit SDHB (Table 2) [16]. *SFXN4 KO* leukemic cells also showed reduced levels of several RC subunits containing ISCs [31].

Whereas no significant change in the activity of the CI, CII and CIV ETC complexes was observed upon SFXN1 gene knockout in HEK cells, CIII activity was dramatically decreased and partially restored upon SFXN1 overexpression [16]. In agreement with the observed decrease in the levels of cytochrome b (MT-CYB), cytochrome b-c1 complex subunit 2 (UQCRC2) and cytochrome b-c1 complex subunit Rieske (UQCRFS1) subunits, Acoba et al. also reported a reduction in CIII2 and in CIII2-CIV subcomplex, whereas the assembly of respiratory supercomplexes was unaffected. Mitochondrial translation is not dramatically impaired in the absence of a functional SFXN1 protein, nevertheless a slight decrease in cytochrome b translation was reported in this study.

No decrease in either the quantity of mtDNA or in the mitochondrial mass was seen in *SFXN1 KO* cells; thus, a general defect in mitochondrial biogenesis can be excluded [8,16]. Current knowledge on Complex III biogenesis is well-described in Reference [40]. Seven assembly factors are implicated in CIII biogenesis in humans (UQCC1-3, CCHL, BCSL1, LYRM7 and TTC19). The Rieske subunit is first translocated from the cytosol to the matrix where it acquires its ISC and is further incorporated in CIII. In the matrix, Rieske is stabilized by the chaperone LYRM7 [41]. BCS1L is required for the translocation of the folded Rieske iron-sulfur protein in the IMM by a mechanism that remains largely unknown [42]. No regulation of the levels of BCSL1 and LYRM7 assembly factors was observed when SFXN1 is absent in mammalian cells [16].

Interestingly, HEK *SFXN1 KO* cells were reported to have markedly reduced levels of Coenzyme Q (CoQ, ubiquinone), a lipid of the IMM that accepts electron from CI and CII and then donates one electron to the ISC of the Rieske subunit and another one to the heme of the cytochrome b of CIII (see Reference [40] and [43] for more details on the transfer of electrons from CoQ to the IMS soluble electron carrier cytochrome c).

Deficiencies of mitochondrial respiration and/or RC activity were also reported for other SFXN, as summarized in Table 2. For example, *SFXN2* knockout led to a decreased activity of CII-CIII and CIV [9]. As no specific impairment in complex III activity has been described nor in *SFXN2* nor in *SFXN4 KO* cells, there is presumably no interaction between those SFXN isoforms and the BCS1L protein (responsible of the GRACILE Syndrome), a mitochondrial chaperone that is anchored to the inner mitochondrial membrane and required for proper Complex III activity [44]. Nevertheless, this possibility cannot be totally discarded, as the patients with S78G point mutation in the *BCS1L* gene have no decreased Complex III activity when compared to other mutations of the same gene.

## 3. Which Place for Sideroflexins in the Regulation of Mitochondrial Metabolism?

### 3.1. Sideroflexins and One-Carbon Metabolism (OCM)

Using CRISPR/Cas9 based-screening, Kory et al. uncovered a function of mitochondrial serine transporter for SFXN1 [8]. The import of serine inside mitochondria is a key step of the OCM, a major metabolic pathway coupled to the synthesis of methyl donors necessary for purine synthesis, epigenetic methylation processes and synthesis of neurotransmitters [46]. Moreover, glycine—arising from serine catabolism by the SHMT2 enzyme [47]—is a key amino acid for the synthesis of heme, a cofactor present in cytochromes of the respiratory chain and other essential proteins, such as CYP450 proteins. Finally, OCM is known as a central pathway ensuring hyperproliferation of cancer cells. Hence, OCM, through the folate cycle, links serine catabolism to purine and nucleotides biosynthesis. Liver, kidney and blood are tissues with high OCM activity, however the role of OCM is not restricted to these organs, but present in all human tissues, including brain [46]. Actually, defective one-carbon metabolism during embryonic development is responsible for neural tube defects.

Whereas Jurkat cells lacking SFXN1 proliferate as wild-type cells do, their proliferation rate is markedly reduced in a medium lacking serine, but is normal in the absence of glycine that can be provided by the catabolism of serine [8]. A lower proliferative rate compared to that of wild-type cells was also reported for HEK *SFXN1 KO* cells in the absence of serine. Interestingly, proliferation of SFXN1 deficient cells was enhanced when formate (OCM metabolite), but not hemin (heme derivative), was added [16]. Additionally, Kory et al. showed that the double knockout of *SFXN1* and *SFXN3* greatly impaired proliferation in a glycine-deficient medium. Apart from human *SFXN4*, overexpression of virtually any *SFXN* family member, including *S. cerevisiae FSF1/YOR271C*, and the two *Drosophila* orthologues *dSfxn1* and *dSfxn2* can rescue the glycine auxotrophy due to the OCM defect induced by the concomitant loss of SFXN1 and SFXN3 in human leukemic cells. However, the defect in purine synthesis is rescued only by SFXN2, SFXN3, dSfxn1 and *S. cerevisiae* FSF1 [8]. Thus, most SFXN appear functionally redundant in serine import, although probably with different kinetic properties. Moreover, they might also ensure the mitochondrial import of other metabolites.

### 3.2. Sideroflexins in Central Carbon Metabolism

Disturbance of central carbon metabolism was reported in SFXN1-null cells. A LC-MS analysis of tricarbolylic acid (TCA) cycle metabolites contained in HEK *SFXN1 KO* cells showed significantly reduced levels of citrate and isocitrate, while α-ketoglutarate (α-KG) was decreased and succinate cellular levels were unchanged [16]. Isotopic labelling experiments helped understanding the role of SFXN1 in mitochondrial metabolism. ^13^C metabolic flux analysis (^13^C MFA) is a useful tool to assess intracellular fluxes and get clues on the metabolic pathways that are differentially activated in mammalian cells depending of the genetic context or environmental conditions [48]. Using ^13^C MFA to investigate metabolic fluxes in HEK *SFXN1 KO* cells, Acoba et al. provided evidence for a reduced activity of the glutamate dehydrogenase (GDH) that converts Glu in α-KG using NAD(P)+ as a coenzyme [49,50]. The lower activity of GDH is unlikely due to a lowering in NAD(P)+ since NAD(P)+/NADPH ratio was unchanged in SFXN1-deficient cells [16]. In animals, GDH is regulated by a wide variety of ligands (NADH, GTP, ATP, palmitoyl-coA, steroid hormones, leucine) and the mitochondrial enzymes SIRT4 and SCHAD. Alanine aminotransferase (ALT) activity is also markedly reduced in SFXN1-null cells [16]. This deficiency in alanine catabolism is probably due to the lower availability of α-KG in SFXN1-null cells. Alanine aminotransferase (also known as GPT) is implicated in L-alanine degradation via the transaminase pathway and uses pyridoxal 5′-phosphate as a cofactor. A comprehensive review of nitrogen utilization and amino acid metabolism can be found in Reference [51]. Mitochondrial levels of GDH and ALT2 (mitochondrial alanine aminotransferase) were not investigated in SFXN1-deficient cells and we wonder if the absence of SFXN1 could trigger a decrease in the mitochondrial import or stability of some mitochondrial enzymes intervening in the catabolism of amino acids that fuel the TCA cycle. Acoba et al. also performed ^13^C MFA with [U-^13^C]-glucose to fuel the TCA cycle with [U-^13^C]-labelled acetyl-coA and provided evidence for an increase in the incorporation of glucose in the TCA cycle [16].

NAD^+^/NADH ratio was also increased in *SFXN1 KO* cells, and altogether, the results obtained by Acoba et al. shed light on a disturbance of central carbon metabolism upon the loss of SFXN1. Whether the deficiency in SFXN1 orthologues and the other human sideroflexins also affects central carbon metabolism is an open question that is not fully elucidated.

## 4. Sideroflexins, Iron Homeostasis and Heme Biosynthesis

### 4.1. A Brief Overview of Iron Homeostasis, ISCs and Heme Biosynthesis

**Figure 4:**
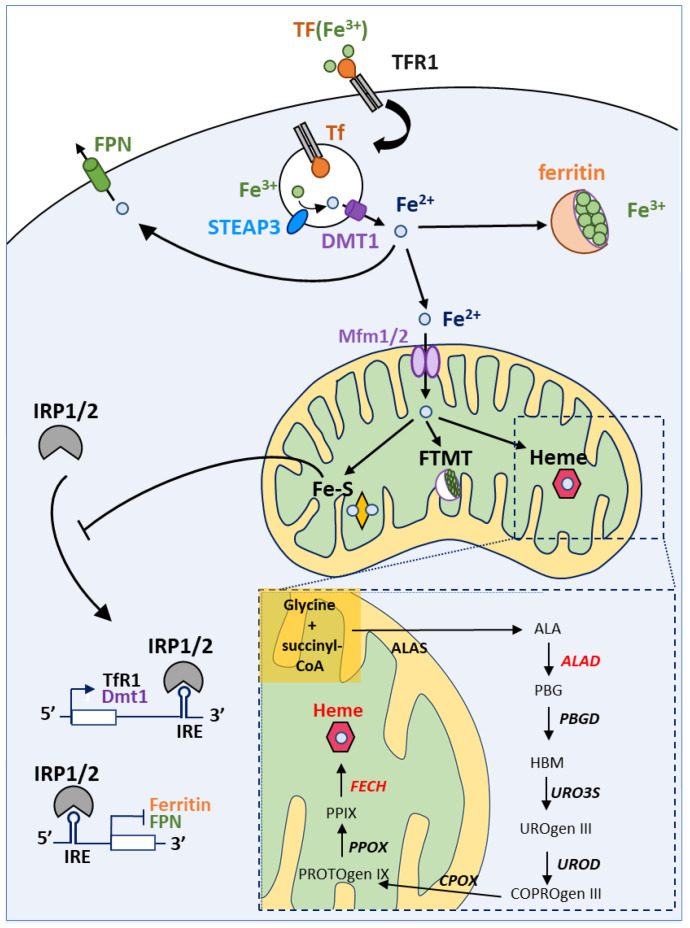
Iron homeostasis and utilization at the cell level. Iron cellular uptake is controlled by transferrin and its receptor (TF and TFR1, respectively). Afterwards, in the endosome, iron is reduced thanks to the action of STEAP3 (which converts the insoluble Fe^3+^ to soluble Fe^2+^) and released from the endosome into the cytoplasm by the DMT1 channel. Free iron can be stored by ferritin in the cytoplasm or can be transported into the mitochondria, thanks to Mitoferrin 1 and 2 transporters (Mfrn1/2). Excess of iron is released out of the cell by Ferroportin (FPN). Inside the mitochondrion, iron can be stored in FTMT (mitochondrial ferritin) or incorporated in heme or Fe-S clusters. IRP1 and 2 (Iron Related Protein 1 and 2) are the major regulators of iron metabolism. In iron-depleted cells, IRP1 can bind IRE (Iron Response Elements) motifs to promote or repress mRNA translation. If IREs are located in the 5′UTR, IRP1 binding represses mRNA translation under low iron levels. On the contrary, transcripts with IREs at the 3′UTR are stabilized and translated upon IRP binding. Hence, low iron levels lead to decreased Ferritin and FPN levels, but promote TFR1 and DMT1 synthesis. High levels of iron prevent IRP1 binding to IREs (see the main text for details).

Iron is an essential cofactor for several enzymes involved in redox reactions due to its ability to exist in two ionic forms: ferrous iron (Fe^2+^) and ferric iron (Fe^3+^). Iron is thus easily oxidized and reduced, making it suitable for redox reactions. Thus, iron is a key player in many important cellular processes, including energy metabolism, respiration and DNA synthesis. The implication of iron in all these processes is done through the incorporation of this atom in complex structures synthesized mainly in the mitochondria: iron-sulfur clusters (ICS) and heme. Iron homeostasis is a tightly controlled process in which numerous proteins intervene [52,53,54,55]. Figure 4 depicts the main actors of iron trafficking and metabolism at the cellular level.

Maintaining iron homeostasis is essential for cell viability and iron intracellular levels are thus tightly controlled by Iron Regulatory Proteins (IRP1/2). IRP1/2 regulate the levels of key proteins intervening in iron homeostasis by binding to Iron Responsive Element (IRE) sequences either located in the 5′ UTR or in the 3′ UTR of mRNA encoding actors of iron metabolism. For example, when cellular iron levels are low, IRP proteins bind to IRE in the 5′ UTR of ferritin and ferroportin mRNAs (among others) and thereby inhibit their translation. The IRPs proteins can also bind to IRE in the 3′UTR of iron-regulated mRNAs, such as TfR1 and DMT1 mRNAs encoding two proteins involved in iron uptake, thereby preventing the endonuclease-mediated degradation of these mRNAs (see Reference [56] for a review). Thus, this regulation by IRP proteins under low iron concentration leads to an increase in iron uptake as well as a decrease in iron storage and export. On the contrary, under high iron levels, the synthesis of iron-sulfur clusters is enhanced. The binding of an iron-sulfur cluster to the IRP1 protein leads a conformational change inhibiting its IRE binding activity but promoting its aconitase activity. The ACO1 enzyme (e.g., Fe-S bound IRP1) catalyzes the conversion of citrate and isocitrate in the cytosol enhancing, probably, NADPH generation and lipid synthesis [57]. Our aim is not to give an extensive review of the IRE-IRP signaling pathway and numerous comprehensive reviews can be found elsewhere, such as in Reference [58].

Iron-sulfur clusters are made up of iron and sulfur ions that come together to form [1Fe-0S], [2Fe-2S], [3Fe-4S] and [4Fe-4S] clusters [59]. Fe-S clusters (ISCs) are found in numerous metalloproteins such as aconitase 1 [54,60,61,62]. Thus, ISCs are involved in a wide variety of cellular processes, among which we can cite the Krebs cycle, mitochondrial respiration, and DNA replication/repair. Assembly of the Fe-S center is carried out by the ISC machinery. Inorganic sulfur is first produced from the cysteine by the cysteine desulfurase NFS1. Then, the Fe-S cluster is formed on the ISC assembly enzyme (ISCU) with the help of frataxin (FXN) [63].

Heme is a complex of ferrous iron and protoporphyrin IX (PPIX). It is an important prosthetic group for many vital proteins, such as hemoglobin, myoglobin, cytochromes and CYP450 proteins [64,65]. Heme is involved in the transport and storage of oxygen, the transfer of electrons for enzymatic redox reactions, signal transduction, ligand binding and control of gene expression [66]. Heme biosynthesis (Figure 4 and Figure 6) is a pathway comprising eight steps, among which four arise inside the mitochondrion (e.g., the first and the last three steps). The rate limiting enzyme of this process is the ALA-synthase (ALAS) responsible for the synthesis of δ-aminolevulinic acid (ALA) from the condensation of glycine and succinyl-CoA, in the presence of pyridoxal-5′-phosphate [67,68]. Two genes encode ALA-synthases: *ALAS1* is the ubiquitously expressed one while *ALAS2* expression is restricted to erythroid cells. Negative feedback regulation of ALAS1 by heme has been reported and will be discussed later. Ferrochelatase (FECH) catalyzes the last step of heme biosynthesis, namely the insertion of iron into PPIX. Heme biosynthesis has been extensively reviewed elsewhere [52,55,69].

### 4.2. Can Sideroflexins Regulate Iron Homeostasis?

The first evidence for a link between sideroflexins and iron metabolism came from a study of the flexed-tail mouse, which harbors a mutation in a locus containing the *Sfxn1* gene [1]. Mice mutant for Sfxn1 displayed sideroblastic anemia, microcytic anemia and hypochromic erythrocytes. Furthermore, flexed-tail mice were also displaying iron deposits in the mitochondria from erythrocyte precursors. Nevertheless, no mechanisms regarding the iron accumulation in the mitochondria were proposed; but since then, sideroflexins were annotated as proteins implicated in iron metabolism.

Based on the annotation of SFXN as transporters of metabolites required for iron metabolism, we and others have tried to monitor the consequences of the loss of SFXN on iron cellular levels. Table 3 summarizes the experimental evidence for an iron imbalance in the absence of SFXN. Whereas Mon et al. reported increased mitochondrial iron levels in HEK *SFXN2 KO* cells [9], an ICP-MS analysis did not show significantly modified cellular or mitochondrial iron levels in HEK *SFXN1 KO* cells compared to parental cells, but an increase in cellular Mn^2+^ [16]. Notable, albeit not significant, it seems that the loss of SFXN1 also slightly enhanced mitochondrial iron levels measured by ICP-MS, with a more pronounced effect in one of the two *SFXN1 KO* clones [16]. Maybe, a significant increase could have been seen with more replicates or by quantifying mitochondrial iron by a TEM-EDX analysis as done for *SFXN4 KO* cells [31]. Despite an appropriate methodology, caution must also be taken when analyzing the results obtained by Mon et al. because this study was done with only one cellular clone obtained after CRISPR/Cas9 invalidation of the *SFXN2* gene. However, expression of a SFXN2-mCherry fusion protein restored basal mitochondrial Fe^2+^ levels in these *SFXN2 KO* cells, as measured with a specific fluorescent probe. Loss of SFXN4 was also proven to alter iron levels in the K562 leukemic human cell line. Whereas the labile cytosolic iron pool was decreased, Paul et al. have provided evidence for a redistribution of cellular iron from the cytosol to the mitochondria in K562 *SFXN4 KO* cells [31].

**Figure 5:**
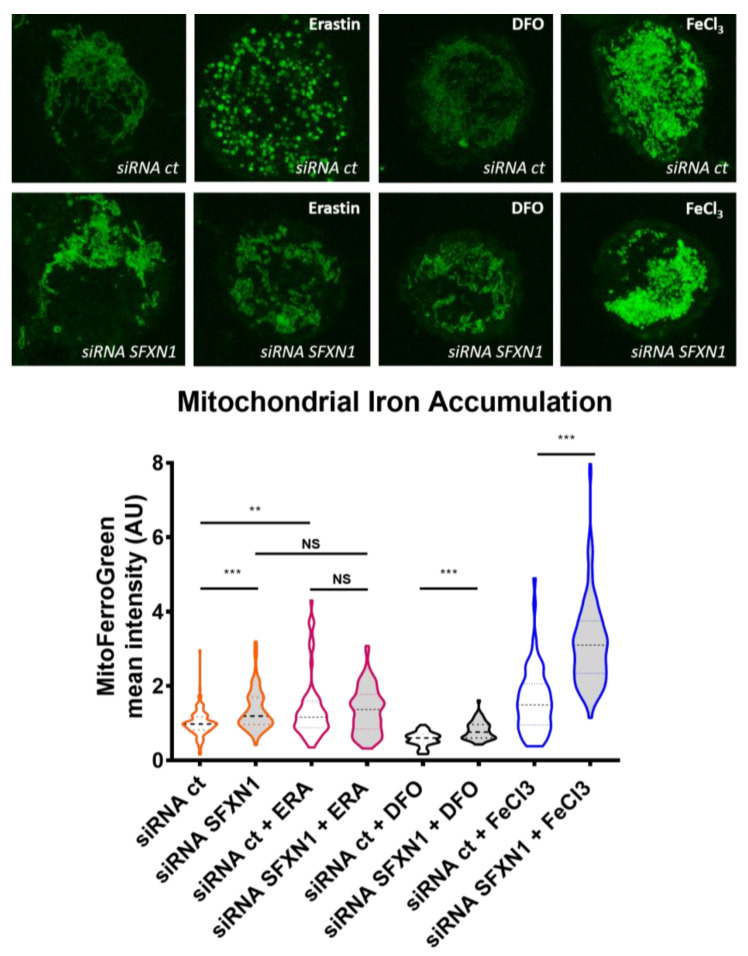
Depleting SFXN1 in HT1080 human cells leads to an intramitochondrial iron accumulation. Top panel: mitochondrial labile Fe(II) staining using the Mito-FerroGreen probe [70] after transient transfection with a control siRNA (siRNA ct) or a pool of SFXN1-targeting siRNA (siRNA SFXN1). Cells were further treated with DMSO (vehicle), erastin, DFO or FeCl_3_. Erastin is a drug that is widely used to trigger ferroptosis, DFO (deferoxamine) is an iron chelator that lowers mitochondrial iron levels and is used as a negative control. FeCl_3_ increases intracellular iron levels and served as a positive control. SFXN1 depleted cells show higher mitochondrial iron levels than control cells (siRNA scramble transfected cells). Same magnification is used for all images and insets from full images taken at 630× are shown here. Bottom panel: quantification of three independent assays (*n* > 50 cells per condition) in which the fluorescent signal is measured and values are normalized to siRNA ct mean levels (mean = 1). After Mann-Whittney tests, significant differences are shown (** *p* < 0.01, *** *p* > 0.001, NS Not Significant). See Appendix A.2. for experimental details.

In our lab, we are interested in the early events triggered by the depletion of SFXN1 in mammalian cells. To investigate the effect of a decrease in SFXN1 protein levels, we chose to transiently deplete SFXN1 in HT1080 human cells using siRNA, and then, we quantified mitochondrial labile Fe(II) levels using the Mito-FerroGreen probe [70]. Depleting SFXN1 in HT1080 cells induced a slight, but reproducible increase in mitochondrial iron levels, as shown in Figure 5. This increase in mitochondrial Fe(II) when SFXN1 levels are lowered, could be either a consequence of a defective heme biosynthesis, since Fe(II) is the substrate of FECH that inserts it into protoporphyrin IX, or a consequence of the catabolism of heme by HO-1 (heme oxygenase-1). Additionally, our data enlighten an erastin-dependent increase in labile Fe(II) mitochondrial levels in HT1080 cells. A similar increase was also reported in erastin-treated MEF cells [71]. Erastin is a small molecule drug commonly used to trigger ferroptosis. As shown in Figure 5, in control cells (siRNA ct), erastin increases mitochondrial iron levels and a punctuate staining is seen, maybe revealing mitochondrial network fission. In SFXN1 depleted cells, erastin does not seem to further increase mitochondrial iron levels. In all conditions, except with erastin, iron levels are increased after SFXN1 depletion, compared to control, suggesting that erastin and SFXN1 could use the same mechanisms to promote an increase in mitochondrial iron levels. In HT1080 and MEF cells, erastin was previously shown to induce *HO-1* expression [71,72], which may explain the increase in mitochondrial Fe(II) that we observed in erastin-treated H1080 cells. Whether reducing SFXN1 levels inhibits FECH activity or promotes heme catabolism must be further investigated.

Altogether, the evidence enounced above point towards a role for SFXN in the maintenance of appropriate iron levels since the depletion or loss of SFXN1, SFXN2 and SFXN4 may increase mitochondrial iron by mechanisms that remain unknown. Mitoferrin1 and Mitoferrin2 are known as iron importers into the mitochondria and ABCB8 as an iron exporter [73]. Thus, due to the fact that iron mitochondrial transporters have been already described, and that the lack of either SFXN 1, 2 or 4 leads to intramitochondrial iron accumulation, we do not favor the possibility that SFXN are iron transporters. Thus, other intriguing possibilities should be explored.

**Figure 6:**
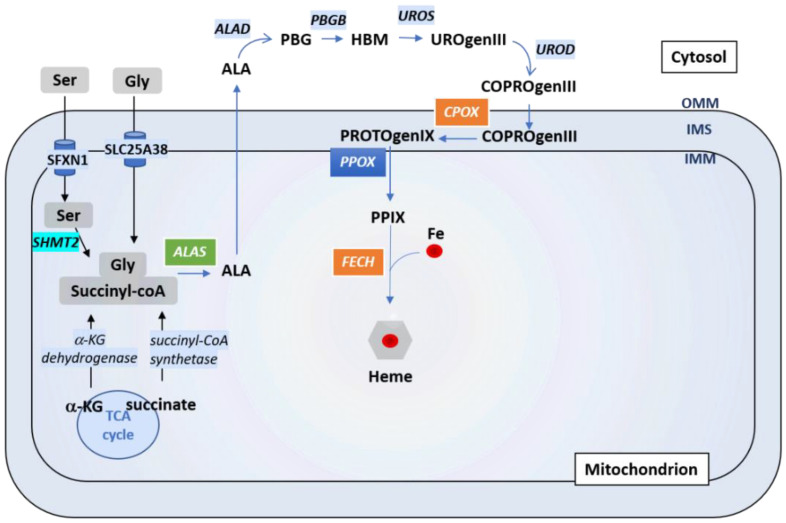
Regulation of heme biosynthesis by SFXN1. Gly and succinyl CoA are the substrates to generate ALA, the first heme precursor, thanks to ALAS enzyme. Gly can enter directly into the mitochondria by SLC25A38, or can be the result of Ser transformation (previously imported by SFXN1) by SHMT2. ALA is further exported to the cytosol where the next steps of heme biosynthesis are catalysed by ALAD, PBGB, UROS and UROD. CPOX, PPOX and FECH are the three mitochondrial enzymes that catalyze the three last steps of heme synthesis (see main text). The last step corresponds to the incorporation of iron into the protoporphyrin PPIX to complete the heme synthesis. Cells lacking SFXN1 show decreased CPOX and FECH mRNA and protein levels (orange box), but a higher amount of ALAS protein (green box), according to Acoba et al. [16].

**Figure A1:**
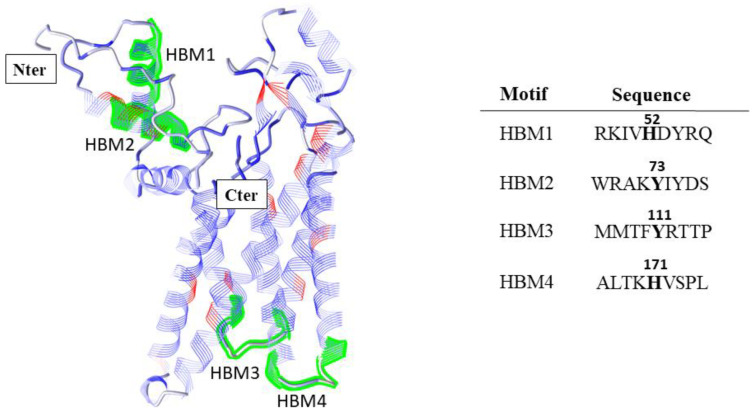
Predicted HBMs in human SFXN1. *Left panel:* The putative HBMs on SFXN1 predicted structure are highlighted (in green). *Right panel*: Sequences of the 9mer motifs in SFXN1 corresponding to predicted HBMs. The position of the potential heme-coordination site (Cys, His or Tyr) is shown (bold).

Proper iron homeostasis requires a fluid transport of iron and its derivatives through the mitochondrial membranes and the cytosol. In this regard, the ALA (Aminolevunilic acid) synthesis requires Gly import through SCL25A38 on the one hand, and ALA export on the other hand, presumably through the same transporter (Figure 6) [74]. SFXN1 was shown to be a Serine transporter in vivo [8]. Intramitochondrial Ser would be catabolized by SHMT2 into Gly and 5,10-meTHF (5,10 methyl tetrahydrofolate) to enter the OCM pathway, necessary for purine synthesis, pointing out that SFXN1 could be linked to the first and limiting step of heme synthesis. Moreover, once protoporphyrin is generated in the intermembrane space, it must enter the mitochondrial matrix for the last heme synthesis step using both ABCB6 and ABCB10 transporters [75,76]. Whether SFXN1 could bind to heme and help in its trafficking is another hypothesis that merits our attention. We thus seek heme binding motifs (HBMs) in SFXN1 with the HeMoQuest tool dedicated to the prediction of heme-coordination sites in protein sequences [77,78] and we found four HBMs that are solvent-accessible (Figure A1, Appendix A). These predicted HBM may permit transient interactions between heme and SFXN1. Biochemical studies are needed to confirm these interactions and further investigate their significance regarding SFXN1 activity.

To conclude, several open questions are remaining about the role of SFXNs in iron homeostasis. For example, are SFXN3 and SFXN5, like SFXN1, 2 and 4, able to regulate iron levels? No studies have been performed in this regard yet. Do sideroflexins alter iron levels by regulating the activity of other regulators implicated in iron homeostasis? How can we explain that low SFXN1 levels (as well as low levels of SFXN2 or 4) lead to an increase of mitochondrial iron, and that an increase in SFXN1 may also trigger an increase in mitochondrial iron levels (see Section 5.2)? What are the relationships between iron homeostasis disturbance and one carbon metabolism? To answer those questions, further work in mammalian cells is needed, and later confirmed using in vivo models.

### 4.3. Which Role for Sideroflexins in Heme Biosynthesis and ISC Biosynthesis?

Whether SFXN1 and its homologues can regulate heme biosynthesis has not been thoroughly investigated so far, but recent studies gave evidence for an impairment of heme biosynthesis when certain members of the SFXN family are lacking [9,16,31]. Interestingly, SFXN1 loss in human kidney embryonic cells was recently reported to impair heme biosynthesis [16]. Indeed, cells lacking SFXN1 showed reduced heme levels, decreased CPOX and FECH transcripts and protein levels, but increased ALAS1 protein levels. It is well-known that heme can induce ALAS1 degradation by a mechanism involving, at least, ALAS1 binding to the mitochondrial protease CplXP [79]. It is thus likely that low heme levels found in SFXN1 cells limits heme binding to ALAS1 and consequently inhibits its degradation by CplXP. These defects in heme biosynthesis may explain the less efficient mitochondrial respiration and, especially, Complex III loss of activity. Accordingly, whereas formate had no effect, hemin supplementation increased CIII activity in wild-type and *SFXN1 KO* cells but only partially restored the assembly of CIII in *SFXN1 KO* cells [16]. However, hemin was unable to restore basal levels of Complex III subunits in HEK *SFXN1 KO* cells suggesting that other defects are present in these cells. Interestingly, DMK (dimethyl- α-KG, a cell permeant analogue of α-KG) rescued, almost totally, CIII subunits levels and CIII activity in HEK *SFXN1 KO*. Succinyl-coA that serves in the first step of heme biosynthesis can originate from α-KG or succinate. Hence, in the mitochondrial matrix, α-KG can be converted in succinyl-coA by α-KG dehydrogenase, a highly regulated enzyme of the TCA cycle [80]. It would thus be interesting to determine if, in addition to the decreased GDH and ALT activity observed in SFXN1 null cells [16], α-KGDH activity is also impaired upon the loss of SFXN1.

Impairment of heme biosynthesis upon SFXN loss could be explained by the function of serine transporter attributed to SFXN. Following its import into the mitochondrion, Ser can be converted in Gly and 5,10-me-THF involved in folate cycle and OCM. An imbalance in the cellular Ser/Gly ratio may impair heme biosynthesis since Gly is (with succinyl-coA) the precursor for the synthesis of protoporphyrins into which iron is incorporated in the final step of heme synthesis catalyzed by FECH (Figure 6). As SFXN1 is presumed to be the mitochondrial transporter of Ser, its loss could increase cellular Ser and lower Gly levels. Indeed, in Jurkat and K562 *SFXN1 KO* cells, the cellular Ser/Gly ratio was increased and associated to increased cellular Ser levels, but the decreased Gly levels [8]. In agreement with an imbalance in serine levels upon SFXN1 loss, HEK *SFXN1 KO* cells also have increased cellular Ser levels and a Ser/Gly ratio, but no decrease in Gly cellular levels were reported [16]. Whether this discrepancy can be explained by a cell type specificity or other reason remains to be elucidated. Notably, mitochondrial levels of those two amino acids have not been assessed and it will be interesting to more specifically address the presence of Ser and Gly inside the mitochondrion by a metabolomics study on this organelle.

SFXN2 has been recently described in HEK293 cells to have a key role in iron metabolism, mainly in heme synthesis [9]. High levels of iron have been shown in mitochondria in *SFXN2* knockout HEK293 cells. Additionally, a decreased activity of Complexes II-IV, but not of the Complex I, was noticed. Complex I subunits contain Fe-S clusters, in contrast to Complex IV, which is mainly composed of heme groups. Complexes II and III contain both Fe-S clusters and heme groups (Figure 2). Thus, as no effect in Complex I was detected, and no decrease in Frataxin (FXN), a mitochondrial enzyme required for the Fe-S cluster formation, nor in ALAS2, the enzyme that catalyzes the first step of the heme biosynthetic pathway, was reported, it was concluded that SFXN2 mutants affected heme synthesis after the first step of heme biosynthesis, but not the Fe-S cluster formation. However, neither the levels of ISC-containing proteins nor those of ALAS1 have been assessed in this study. It is surprising because ALAS2 is the erythroid specific form and ALAS1 the housekeeping one.

We propose a few possibilities to explain the *SFXN2* knockout cells phenotype. The lack of SFXN2 could either lead to an impaired ALA export or no mitochondrial import of protoporphyrin (PPIX) for the last step of the heme pathway. A defective mitochondrial export of the heme group is another plausible explanation. Finally, other options could be possible as an interaction of SFXN2 with BCS1L, a chaperone anchored to the inner mitochondrial membrane that is required for the proper assembly of Complex III (see Section 2.2 for more details). In all those cases, an intramitochondrial iron accumulation is presumed. All those possibilities, and others, must be studied to be able to clarify the possible role of SFXN2 in heme biosynthesis.

### 4.4. Which Role for Sideroflexins in ISC Biosynthesis?

Loss of SFXN also seems to impair ISC biogenesis. Indeed, in the absence of SFXN4, there is a decrease in Fe-S cluster levels, which is consistent with the decrease of Complex I activity seen in *SFXN4 KO* cells, pointing out that this SLC56 carrier could play a role in Fe-S biosynthesis [30,31]. As a consequence of the low Fe-S levels, IRP1 aconitase activity, as well as labile iron cytosolic levels, also decreases, whereas mitochondrial iron increases, suggesting that iron import in the mitochondria is not impaired, and instead possibly enhanced. Those features are very similar to the lack of mitochondrial frataxin, which leads to Friedreich’s Ataxia, also known as X-linked sideroblastic anemia. Frataxin (FXN) is a mitochondrial chaperone that interacts with aconitase in a citrate-dependent manner to convert (3Fe-4S)1+ inactive enzyme into a [4Fe-4S]2+ active one within the Krebs cycle. It also interacts with the ISCU-NFS1 (Iron-Sulfur Cluster Scaffold-Cysteine desulfurase) in the final steps of Fe-S formation [81,82]. The reduction of mitochondrial aconitase (ACO2) in *SFXN4 KO* cells [31] suggests that SFXN4 could participate in the Fe-S biosynthesis, maybe through an interaction with Frataxin (FXN). It has been previously reported that FECH, an important enzyme for heme biosynthesis, Mfrn1, an iron transporter into the mitochondria, and ABCB10, a protoporphyrin IX transporter, could form a complex in mouse erythroleukemia (MEL) cells to direct iron incorporation into protoporphyrin to form heme [54,83]. Taken together, those results open the possibility that SFXN4 and FXN interact with other proteins such as aconitase or the ISCU-NFS1 multimeric complex to maturate the Fe-S clusters. We have recently performed a screen with the aim of identifying the direct partners of SFXN1 protein in MCF7 cells (Tifoun et al. in preparation), and even though SFXN1 does not interact directly with FXN, it is still possible that SFXN4 could do so. In SFXN44 mutants Fe-S synthesis is reduced, pointing out that SFXN4 may play a role in the first steps of Fe-S cluster formation, maybe through FXN interaction. A recent study shows that the ISC (Iron Sulfur Cluster, composed by NFS1, ISCU and FXN) function requires L-Cysteine to generate disulfide groups necessary to form the Fe-S clusters [84]. Moreover, it has been postulated that SFXN1 could transport not only serine, but alanine and possibly also glycine and cysteine in vitro [8]. Actually, in SFXN1, depleted cells have a proliferative advantage in media containing low cystine (dimer of cysteine formed under oxidant conditions), this could be due to the fact that the amino acid cysteine is necessary for cytosolic glutathione synthesis and that a loss of mitochondrial import would increase its availability for those purposes [8]. The lack of SFXN1 activity can be overcome by SFXN2 and SFXN3, but not by SFXN4 [8]. SFXN4 cannot substitute SFXN1 for Ser import into the mitochondria, but it could maybe have a higher affinity for Cys. This may explain why SFXN1 and SFXN2 mutants present mainly problems in heme synthesis, whereas *SFXN4 KO* cells have deficiencies in Fe-S cluster formation, as Ser and Gly are essential for the ALA synthesis and Cys is required for proper Fe-S maturation.

How could SFXN regulate iron levels and heme biosynthesis remains unanswered and whether SFXN impair mitochondrial iron and heme homeostasis by direct or indirect actions is unknown. We have recently documented the interaction between SFXN1 and ATAD-3 (Tifoun et al. in preparation). Because *Caenorhabditis elegans* ATAD-3 was shown to modulate mitochondrial iron and heme homeostasis, heme biosynthesis regulation by SFXN1 may depend on its interaction with ATAD-3. Interestingly in *atad-3* (RNAi) worms, mitochondrial but not cytosolic iron levels were increased and an altered expression of iron homeostasis genes was reported [85]. Indeed *atad-3* knockdown (KD) led to an increase in *ftn-1*, but a decrease in *ftn-2* mRNA (respectively encoding the intestinal ferritin heavy chain and a more ubiquitous one). *aco-1* (encoding the homologue of the mammalian IRP responsible of the post-translational regulation of ferritin), *fpn-1.1* (encoding a *C. elegans* ferroportin homologue) and *smf-3* mRNA (involved in the cellular uptake of non-heme iron) were reduced. Expression of *mfn-1* (the sole Mitoferrin encoding gene in *C. elegans*) was unchanged upon *atad-3* knockdown. In agreement with a mitochondrial iron overload, *atad-3* KD in worms also led to an accumulation of Hemin (a heme-containing protein involved in erythroid differentiation) and a fluorescent analogue of heme.

Interestingly, a new mutation of ATAD3A (Arg528Trp), which has been described in seven families [86], is responsible of developmental delay, hypotonia, optic atrophy, axonal neuropathy and hypertrophic cardiomyopathy. In some of those individuals, a deficiency of complex III and citrate synthase was detected. Those results look similar to the consequences of the lack of SFXN1 or SFXN4 proteins. ATAD3A, being a transmembrane protein that binds both external and internal mitochondrial membranes, could interact with SFXN1 and/or SFXN4 to control iron metabolism. Moreover, the use of *Drosophila* in this study, allowed to see that either lack of *bor* (*belphegor*, *ATAD3A* homologue), either the expression of a R534W form, a variant of Arg528Trp human ATAD3A, in the larval neuromuscular junctions (NMJ) promoted a decrease in mitochondrial content, aberrant mitochondrial morphology and increased autophagy. Complementary, *bor* overexpression promoted larger and elongated mitochondria in the NMJ. Whether the SFXN family has a role in autophagy remains completely unexplored and merits attention.

## 5. Sideroflexins, Ferroptosis and Ferritinophagy

### 5.1. SFXN, Cell Death and Ferroptosis

Growing evidence supports the key role of iron metabolism in ferroptosis, even if the exact mechanisms are not fully elucidated [87]. Ferroptosis is a physiological cell death contributing to tissue homeostasis and implicated in pathology (cancer, neurodegenerative disease and cardiac injury). Mechanistically, ferroptosis is an iron-dependent, but caspase-independent regulated cell death (RCD) triggered by uncontrolled lipid peroxidation leading to dramatic morphological changes in mitochondria. For recent reviews on the place of mitochondria in ferroptosis regulation, the reader is invited to refer to [88,89]. Ferroptosis can be triggered by diverse drugs such as erastin, RSL3 or FIN56, among many others, and this type of RCD is prevented by iron chelators and antioxidants [90]. The mitochondrion appears as a main contributor to ferroptosis because of its central place in iron metabolism and the fact that several mitochondrial metabolic pathways—including the TCA cycle and ETC—contribute to PL-PUFA (polyunsaturated fatty acid containing membrane phospholipids) peroxidation.

Little data are available on the role of SFXN in cell death and ferroptosis regulation. *SFXN4* gene knockout was reported to promote cell death of K562 human cells in galactose-containing medium, together with an increase in caspase 3/7 activity [31]. Whether the loss of SFXN4 triggers ferroptosis was not investigated to our knowledge. Interestingly, in HEK kidney embryonic cells, *SFXN2* gene knockout seems to sensitize cells to erastin-induced cell death; however, the underlying mechanisms were not deeply investigated [9].

Recently, SFXN1 was shown to participate in LPS-induced ferroptosis in H9c2 cardiomyocytes, a process depending of NCO4A-mediated ferritinophagy [91]. Li et al. showed an LPS- and NCOA4-dependent upregulation of SFXN1 and documented the role of SFXN1 in LPS-induced ferroptosis. Briefly, in LPS-treated H9c2 cardiomyocytes cells, knockdown of SFXN1 increased cell viability, restored intramitochondrial iron basal levels, inhibited mitochondrial ROS production, decreased lipid peroxidation and levels of PTGS2 (also known as cyclooxygenase-2) and MDA. Collectively, these data suggest that SFXN1 promotes LPS-induced ferroptosis, however the molecular mechanisms are far from being clear. Li et al. explained this role by SFXN implication in the iron mitochondrial import, which has not been proven yet. Further work is thus needed to investigate the relationships between SFXN1 and ferroptosis, and the precise mechanisms whereby SFXN1 could regulate iron levels and cell death. It will also be interesting to determine if SFXN1 mediates LPS-induced ferroptosis in other cell types, as well as its implication in ferroptosis mediated by different inducers (such as erastin, RSL3, FIN56 or other drugs). As Acoba et al. reported lowered CoQ levels in *SFXN1 KO* cells and CoQ is an antioxidant and a cofactor for the ferroptosis suppressor FSP1 [92], we expect that an imbalance in SFXN1 levels may favor ferroptosis through a direct or indirect regulation of CoQ levels. It would thus be interesting to study FSP1 activity in *SFXN1 KO* cells.

### 5.2. SFXN1 and Ferritinophagy

To limit the toxicity of free Fe^2+^, molecular traps—e.g., Ferritin and FtMt (mitochondrial ferritin)—exist in the cytosol and the mitochondrion, respectively, as stated earlier. Ferritinophagy, the lysosome-dependent mechanism whereby iron is mobilized from ferritin, can also contribute to ferroptosis induction. In this process, the selective cargo receptor NCOA4 (nuclear receptor coactivator 4A) binds to ferritin and targets this iron storage protein to the lysosomes, thus promoting ferritin degradation and the subsequent release of iron [93]. In apelin-13 induced cardiomyocytes hypertrophy, Tang et al. recently reported a decrease in FTH (ferritin heavy chain), together with an upregulation of NCOA4 and SFXN1 [94]. Immunohistochemical analysis of hypertrophic heart tissue also highlighted an upregulation of NCOA4 and SFXN1. The siRNA-mediated depletion of NCOA4 restored basal levels of SFXN1 in cardiomyocytes, suggesting that apelin-13 mediated upregulation of SFXN1 could depend on NCOA4. In the presence of apelin-13, the knockdown of SFXN1 decreased iron overload and mitochondrial ROS production in ferric ammonium citrate—treated cardiomyocytes. How NCOA4 could upregulate SFXN1 remains unanswered, as well as the role of SFXN1 and the other SFXN/SLC56 transporters in cardiac hypertrophy. In this study, SFXN1 is proposed to be an iron importer, together with mitoferrin 1 and 2, which are upregulated. The increase of mitochondrial iron in the induced cardiomyocytes hypertrophy model responds to the elevated SFXN1 levels, and higher amounts of iron would promote ROS production thanks to the Fenton reaction, an increase of lipids peroxidation and finally, an induction of ferroptosis. Nevertheless, the mechanisms that allow SFXN1 to control iron levels are not addressed nor whether SFXN1 is the most important player in regulating mitochondrial iron, aside from mitoferrins and ferritin, is discussed.

NCOA4 mediated regulation of SFXN1 was also reported in a recent study addressing the role of ferritinophagy in sepsis-induced cardiac injury [91]. In this study, SFXN1 was shown to be upregulated at the mRNA level in LPS-treated cardiomyocytes, but whether this upregulation results from a transcriptional activation or an enhanced stability of mRNA was not studied. To date, the regulation of SFXN expression has not been deeply investigated and further work is needed to document this point. However, intracellular iron may be important for NCOA4-mediated SFXN1 regulation since the iron chelator deferoxamine (DFO) was shown to decrease LPS-induced SFXN1 accumulation [91]. Li et al. used immunofluorescence to show this DFO-mediated downregulation of SFXN1 and this must be confirmed using Western blot.

**Figure 7:**
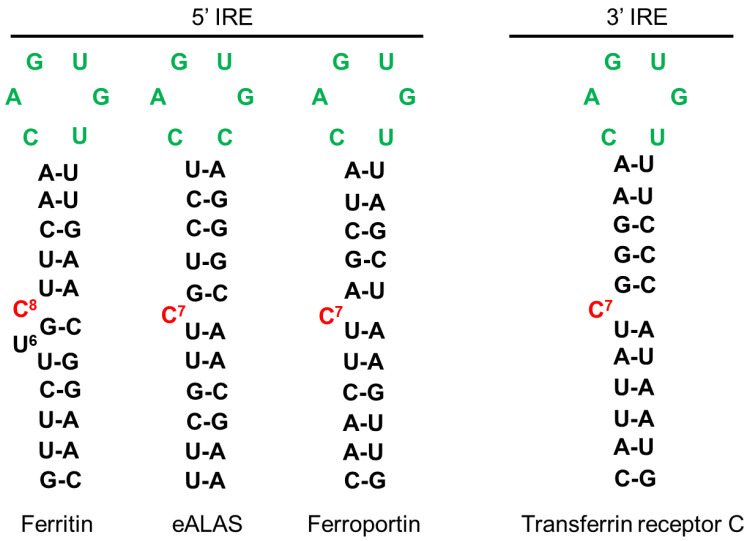
IRE sequences from known proteins involved in iron metabolism. IRE sequences can be localized at 5′ or 3′. In the absence of iron, IRP1 binds the sequences located at 5′ of blocking the translation of the RNA. Ferritin, ALAS and Ferroportin are proteins involved in iron storage, heme synthesis and iron export, respectively. In the same situation, IRP binding to 3′ sequences, stabilizes the RNA promoting the translation of, for example, Transferrin receptor, involved in iron import. In the opposite situation, with high iron levels, IRP binds to iron, which unbinds the IREs, thus promoting translation of Ferritin, ALAS and Ferroportin and leading to Transferrin receptor RNA decay, which is no more protected by IRP1. Green nucleotides form the six-nucleotide apical loop of IRE.

**Figure 8:**
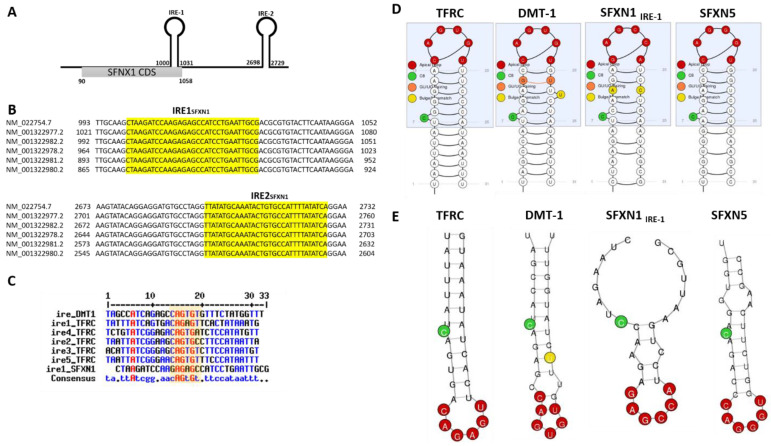
Predicted IRE in SFXN transcripts. (**A**). Two IREs were found in the 3′UTR of SFXN1 transcripts using the SIREs Web Server 2.0. The first one is located at the end of the coding sequence. (**B**). Alignment showing the position of the two IREs in SFXN1 transcripts. All except one shorter SFXN1 transcript variant possess putative IREs. (**C**). Alignment of the IREs of DMT-1, transferrin receptor (TFRC) and SFXN1 transcripts using MultiAlin. The consensus highlights the position of the six-nucleotide apical loop (5′-CAGWGH-3′) as shown in the yellow box. *D*, *E*. Schemes (**D**) and RNA fold prediction (**E**) for the IREs from TFRC, DMT-1, SFXN1 and SFXN5 transcripts generated by the SIREs Web Server 2.0.

The iron-mediated regulation of SFXN1 levels is intriguing and we wondered if iron could regulate translation or mRNA stability by IRP-dependent molecular mechanisms. We hypothesize that IRP proteins, which are major regulators of iron homeostasis acting at the post-transcriptional levels, could modulate SFXN levels through binding to cis-regulatory IRE response elements in SFXN1 transcripts. We thus searched for IRP-binding sites in SFXN transcripts. The IRE found in some of the iron-regulated transcripts are shown in Figure 7. Canonical IRE are motifs composed of a six-nucleotide apical loop (5′-CAGWGH-3′) [95]. Using an IRE prediction tool (“SIREs Web Server 2.0” (http://ccbg.imppc.org/sires/) [96], we retrieved putative IRE in all human SFXN1 variant transcripts except for one (Table 4). One IRE of high quality and a second one of low quality are found, respectively, at the end of the SFXN1 coding sequence and in the 3′ UTR (Figure 8). Additionally, human SFXN2 transcripts possess one putative medium-quality IRE and SFXN5 transcripts contain a putative high-quality IRE, at their 3′ UTR. Interestingly, no IREs are predicted neither in SFXN3 nor in SFXN4 mRNAs. As SFXN1 and SFXN3 are closely related and seem to have highly similar three-dimensional structure, it is tempting to hypothesize that they can be differentially regulated depending on iron levels. In *Drosophila*, no putative IREs are predicted in any of the two mRNAs encoding dSfxn1/3 and dSfxn2, the SFXN orthologues found in flies. The presence of putative IREs at the 3′UTR of some of SFXN transcripts is suggestive of their IRP-mediated stabilization. We thus expect an increase of SFXN1 levels under low iron levels, when IRP1 lacks its Fe-S cluster and IRP2 is degraded. The latter is not in agreement with the DFO-mediated downregulation of SFXN1 levels reported by Li et al. [91]. IRE motifs found in SFXN transcripts are non-canonical IRE motifs derived from IRE sequences identified in IRP-interacting mRNAs uncovered in the genome-wide SELEX experiments [97,98,99]. Having found IRE in SFXN1 transcripts is in favor of an iron-mediated regulation of SFXN levels, however, whether the IREs found in SFXN1, SFXN2 and SFXN5 transcripts are functional, is a point that needs to be further investigated.

To conclude, despite two works that started to shed light on SFXN1 role in iron homeostasis and ferroptosis [91,94], how this metabolite transporter exerts its function is far from being clear and more work is required to properly elucidate, mechanistically, how SFXN1 is implicated in iron homeostasis.

## 6. Sideroflexins in Aging: May SFXN Regulate Neuronal Physiology and Retinal Function?

In this part, we discuss the potential role of SFXN in neuronal pathophysiology, aging and retinal function.

### 6.1. Sideroflexins and Biometals in Neuronal Physiopathology

Collectively, neurodegenerative diseases constitute a major public health concern. As an example, in the European Union, the number of people living with dementia is estimated to be around eight million according to a recent report from Alzheimer Europe and this number is set to double by 2050. Better knowledge of the molecular pathways favoring neurodegeneration is thus needed to propose new therapeutic avenues to counteract Alzheimer’s or Parkinson’s disease progression.

Brain accumulation of biometals—including iron and manganese—has been observed in neurodegenerative diseases and associated with a decline in cognitive functions [100,101,102]. Accumulation of biometals can be detrimental and may promote protein aggregation. Hence, Amyloid beta peptide (Aβ), which forms toxic aggregates in the brain of patients who suffered from Alzheimer’s disease, is known to interact with iron [103,104,105,106]. Aβ toxicity was reported to be suppressed by the iron storage protein Ferritin in *Drosophila* [107].

We postulate that some SFXN may share a neuroprotective role because SFXN are present in brain neurons (Human Protein Atlas, Reference [18] and our unpublished data) and a decreased expression of SFXN1 and SFXN3 was linked to Alzheimer’s and Parkinson’s disease (AD and PD). Indeed, SFXN1 is decreased in brains of AD patients [108] and SFXN3 downregulated in late stage PD dopamine neurons from *substantia nigra* [109]. Additionally, P-element heterozygous disruption of the *Drosophila* gene encoding dSfxn1/3 enhanced tau toxicity in a *Drosophila* model commonly used to study neurodegeneration [108]. Under physiological conditions, SFXN3 and alpha-synuclein (α-Syn, a PD marker protein) levels were inversely correlated in a murine model, whereas overexpressing *dSfxn1/3* impaired synapse morphology at the *Drosophila* neuromuscular junction [45]. It is tempting to link a putative iron-dependent regulation of SFXN, as discussed above, and the known regulation of α-Syn by IRPs. Hence, an IRE is found in the 5′ UTR of α-Syn mRNAs and IRP-mediated translational inhibition is relieved upon high iron levels [58,110]. This could explain the opposite regulation of α-Syn and SFXN levels. However, we did not find putative IRE in SFXN3 transcripts, as stated above.

Decreased levels of SFXN1 in the hippocampus were also observed in a rat model with bilateral ovariectomy displaying depressive behaviors and cognitive impairment [111]. Recent evidence points towards a regulatory role of SFXN in iron homeostasis/utilization at the cellular level [9,16,31]. However, iron homeostasis and heme biosynthesis have not been investigated specifically in SFXN-deficient neurons yet, and it would be interesting to question this point. Besides the mitochondrial accumulation of iron reported when some SFXN are lacking, Acoba et al. also reported a decrease in manganese levels in SFXN1-null cells [16]. Manganese is an essential metal element required for the activity of certain enzymes (such as MnSOD) and both insufficiency and overexposure can affect neuronal physiology and cognitive functions [112]. Thus, SFXN might regulate neuronal physiology in participating in biometals homeostasis.

Whether SFXN are able to regulate ferroptosis is also an important concern, because ferroptosis is one of the most important regulated cell death in brain [113]. Ferroptosis was reported in Parkinson’s disease, Alzheimer’s disease and Huntington’s disease and other neurologic disorders. Using in vitro, ex vivo and in vivo (mouse) PD models, Do Van et al. [114] reported ferroptosis in PD dopaminergic neurons, a process that was reversed by Ferrostatin-1, a selective inhibitor of erastin-induced ferroptosis, which inhibits lipid ROS. Growing evidence also highlights the implication of ferroptosis in Alzheimer’s disease [115], a neurodegenerative disease characterized by cognitive functions and memory impairment, synaptic loss and neuronal cell death. In mouse, conditional deletion in forebrain neurons of glutathione peroxidase 4 (Gpx4) gene altered cognitive functions (spatial learning and memory) and triggered hippocampal neurodegeneration with hallmarks of ferroptosis [116].

To conclude, further investigations must be undertaken to precisely specify the role of SFXN1 and its homologues in brain biometals homeostasis and neurodegeneration.

### 6.2. Sfxn and Retinal Degeneration

Iron levels vary during retina development, with gender and it accumulates during aging. When supply does not equal demand (e.g., if retinal blood flow is impaired), retinal neurons are at risk of excitotoxic cell death and vision is impaired or lost [117,118].

Many proteins are involved in iron homeostasis in the retina, and most of the rodent models studied, are related to human pathologies, like human atransferrinemia (lack of transferrin), hemochromatosis type IV (lack of ferroportin) or microcytic hypochromic anemia with iron overload (decrease in DMT1), among others (see [118] for a review). Human transferrin electrotransfection in rodents was shown to protect retinal structure and function, reducing microglial infiltration and preserving the integrity of the outer retinal barrier in a photo-oxidative model. Transferrin, a natural iron chelator, delayed also the retina degeneration and decreased oxidative stress [119]. This work validates iron overload as a therapeutic target for pathologies as retinitis pigmentosa or age-related macular degeneration. Taking into account the relationships between SFXN and iron metabolism, we expect that the loss of SFXN could impair retinal function. Accordingly, in mice, Sfxn3 mutations lead to retinal degeneration [120]. Using forward genetics and screening by optical coherence tomography, Chen et al. identified the *pew* and *basilica* mutations in the *Sfxn3* gene, leading to a significant decrease in the outer retina thickness. Mice with CRISPR-Cas9-induced Sfxn3 loss-of-function mutations were further generated to investigate the consequences on the retinal structure and function. Mice with predicted dramatically shortened Sfxn3 proteins showed retinal impaired morphology (decreased retinal thickness, especially that of the outer retina, and loss of the hexagonal shape of retinal pigmentary epithelium cells) and abnormal fundus and vasculature compared to controls. Retinal thickness even decreased with age in favor of a retinal degeneration due to the lack of functional Sfxn3. Whether those defects are linked to an impairment in iron homeostasis was neither explored nor discussed. In our opinion, SFXN3 may regulate intracellular iron levels, thus protecting the retina from oxidative stress. Moreover, in humans, SFXN4 loss-of-function is associated with optic atrophy [19,21], pointing to SFXNs as a central family of proteins required for proper retina development and homeostasis.

## 7. Conclusions and Open Questions

SFXN/SLC56 is a new family of mitochondrial proteins that have important roles in amino acid transport and in iron homeostasis. Several studies associate SFXN depletion with an increase in mitochondrial iron, deficiencies in carbon metabolism and RC activity and ferroptosis, in cell culture, in animal models and in human pathology, thus making the SFXN an interesting target for tissue degeneration therapy. However, even though those links seem to be clear and reproducible, nothing is known about the mechanism of action of SFXN. Do all the isoforms have the same functions (different members are expressed in different tissues)? As there are several transcripts for each isoform, do those different transcripts generate different proteins with different kinetic properties? If SFXN are not iron transporters, how can they control mitochondrial iron levels? How can they control mRNA and/or protein levels of some key heme regulators (CPOX, FECH and ALAS)? Some SFXN present putative IRE, but others do not; are all SFXN sensitive to iron content and to IRP1/2 regulation?

We think that a better knowledge on SFXN biochemistry is needed to properly decipher the functions of each SFXN member, to know whether they all have redundant functions, their interaction with other proteins or with other SFXN, and how they are regulated.

## Figures and Tables

**Table 1:** Evidence for a mitochondrial localization of Sideroflexins.

SFXN	Model	Localization	Experiment	Reference
SFXN1	Mouse	IMM	Co-fractionation	Fleming et al. 2001 [1]
	Human cells (Jurkat, K562)		Immunoblot on affinity-purified mitochondria STED (co-localization of Flag-SFXN1 and COX4)	Kory et al. 2018 [8]
	Human cells (MCF7, HT1080), *Drosophila*		Immunoblot on mitochondrial extracts (fractionation) Confocal microscopy, Proteomics (LC-MS/MS on SFXN1 IP)	Our unpublished data
	Human cells (HEK)		SILAC-based proteomics coupled LC-MS/MS, carbonate extraction, digitonin fractionation	Acoba et al. 2020 [16]
SFXN2	Human cells (HeLa)	OMM or IMM	Confocal microscopy (Tom20 co-localization)	Mon et al. 2018 [9]
	Human cells (Jurkat, K562)		Immunoblot on affinity-purified mitochondria	Kory et al. 2018 [8]
	Human cells (HEK)		SILAC-based proteomics coupled LC-MS/MS	Acoba et al. 2020 [16]
SFXN3	Rat embryonic brain cells	IMM	Fractionation, Confocal microscopy (co-localization with COX4), TEM	Rivell et al. 2019 [18]
	Human cells (Jurkat, K562)		Immunoblot on affinity-purified mitochondria	Kory et al. 2018 [8]
	Human cells (HEK)		SILAC-based proteomics coupled LC-MS/MS	Acoba et al. 2020 [16]
SFXN4	Human cells (HeLa)	IMM	Fractionation and protease protection assay	Hildick-Smith et al. 2013 [19]
	Human cells (Jurkat, K562)		Immunoblot on affinity-purified mitochondria	Kory et al. 2018 [8]
	Human cells (HEK)		SILAC-based proteomics coupled LC-MS/MS	Acoba et al. 2020 [16]
SFXN5	Human cells (HEK) Mouse astrocytes, human cortex and spinal cord		SILAC-based proteomics coupled LC-MS/MS Immunocapture of GFP-OMM-tagged mitochondria (MitoTag mice), immunostaining	Acoba et al. 2020 [16] Fecher et al. 2019 [20]

IMM: inner mitochondrial membrane, IP: immunoprecipitation, OMM: outer mitochondrial membrane, STED: stimulated emission depletion, TEM: Transmission Electron Microscopy, SILAC: Stable isotope labelling of amino acids, LC-MS/MS: Liquid chromatography and tandem mass spectrometry.

**Table 2:** Consequences of sideroflexins (SFXN) deficiency on the electron transport chain (ETC) complexes.

SFXN	Model	Complex	Data	Reference
SFXN1	HEK *SFXN1 KO* cells HeLa *SFXN1 KO* cells	CI	No significant loss of activity SDHB ↓	Acoba et al. 2020 [16]
		CII	No significant loss of activity UQCRC2 ↓↓ UQCRFS1 ↓↓	
		CIII	Cytochrome b ↓↓↓ Significant loss of activity Reduced levels of CIII_2_ and CIII_2_-CIV respiratory complexes	
SFXN2	HEK *SFXN2* KO cells	CI CII-CIII CIV	No significant loss of activity Significant loss of activity Significant loss of activity	Mon et al. 2019 [9]
SFXN3	*SFXN3 KO* mouse	CI, CIV	No significant loss of activity	Amorim et al. 2017 [45]
SFXN4	Primary fibroblasts from two individuals with *SFXN4* mutations	CI + CIII	Decreased activity	Hildick-Smith et al. 2013 [19]
	*SFXN4 KD* zebrafish	CI CI + CIII	Decreased activity	Sofou et al. 2019 [30]
	K562 *SFXN4 KO* cells	CI CII CIII CIV	NDUFB8 ↓ SDHB ↓ UQCRC2 ↓ COX2 ↓	Paul et al. 2019 [31]
SFXN5			N.A. ^1^	

↓ indicates decreased levels of respiratory complexes subunits (↓ low, ↓↓ medium, ↓↓↓ high); ^1^ N.A.: Not addressed.

**Table 3:** Regulation of systemic or cellular iron levels by SFXN.

Protein	Model	Evidence	Methodology	Reference
SFXN1	Mouse	Iron overload in mitochondria of erythrocytes in the *flexed-tail* mouse	Iron mitochondrial staining	Fleming et al. 2001 [1]
	HEK *SFXN1 KO* cells	Increased mitochondrial iron	ICP-MS	Acoba et al. 2020 [15]
SFXN2	HEK *SFXN2 KO* cells	Increased mitochondrial iron levels	ICP-MS Mito-FerroGreen staining and confocal microscopy	Mon et al. 2019 [9]
SFXN3	Mouse *Sfnx3* KO	Decreased circulating iron levels in male transgenic mice homozygous for the Sfxn3^tm1b(KOMP)Wtsi^ allele	Biochemical assay	The IMPC database ^1^
SFXN4	K562 *SFXN4 KO* cells	Decreased labile iron pool	Indirect biochemical measure based on the dequenching of calcein upon release of iron TEM-EDX	Paul et al. 2019 [31]
		Increased mitochondrial iron levels		
SFXN5		no data available	-	-

ICP-MS is for inductively coupled plasma atomic emission—mass spectrometry, TEM-EDX is for Transmission electron microscopy—Energy dispersive X-ray analysis. ^1^ website page for SFXN3: https://www.mousephenotype.org/data/genes/MGI:2137679#phenotypesTab.—means no data available.

**Table 4:** Location of predicted IRE in SFXN1 splicing variants.

Sequence ID	mRNA Length	CDS Position	Product	IRE Position
NM_022754.7 Homo sapiens sideroflexin 1 (SFXN1), transcript variant 1, mRNA	4066	90–1058	sideroflexin-1 isoform 1	1000–1031 2698–2729
NM_001322977.2 Homo sapiens sideroflexin 1 (SFXN1), transcript variant 2, mRNA	4094	118–1086	sideroflexin-1 isoform 1	1028–1059 2726–2757
NM_001322978.2 Homo sapiens sideroflexin 1 (SFXN1), transcript variant 3, mRNA	4037	244–1029	sideroflexin-1 isoform 2	971–1002 2669–2700
NM_001322980.2 Homo sapiens sideroflexin 1 (SFXN1), transcript variant 4, mRNA	3938	90–875	sideroflexin-1 isoform 4	872–903 2570–2601
NM_001322981.2 Homo sapiens sideroflexin 1 (SFXN1), transcript variant 5, mRNA	3966	118-903	sideroflexin-1 isoform 4	900–931 2598–2629
NM_001322982.2 Homo sapiens sideroflexin 1 (SFXN1), transcript variant 6, mRNA	4065	272–1057	sideroflexin-1 isoform 2	999–1030 2697–2728
NM_001322983.2 Homo sapiens sideroflexin 1 (SFXN1), transcript variant 7, mRNA	959	90–818	sideroflexin-1 isoform 3	No IRE

## Data Availability

The data that support the findings of this study are available from the corresponding author, N.L.F., upon reasonable request.

## Abbreviations

ABCB6: ATP Binding Cassette Subfamily B Member, ACO1: Aconitase1, AD: Alzheimer’s disease, α-KG: α-ketoglutarate, α-KGDH: α-ketoglutarate deshydrogenase, AGK2: AcylGlycerol Kinase, ALA: Aminolevulinic acid, ALAD: Aminolevulinic acid deshydratase, ALAS: Aminolevulinic acid synthase, ALT2: Alanine aminotransferase 2, ICP-MS: Inductively coupled mass spectrometry, a-Syn: Alpha-synuclein, ATAD-3: ATPase family 3A domain containing protein, Aβ: Amyloid beta peptide, BBG-TCC: Brain Bergmann Glial cell- Tricarboxylate carrier, BCS1L: Ubiquinol-Cytochrome C Reductase Complex Chaperone, BMP: Bone Morphogenetic Protein, CCHL: Cytochrome c heme lyase, COPROgenIII: Coproporphyrinogen III, CoQ: Coenzyme Q, COX4: Cytochrome c oxidase subunit 4, COXPD18: Combined oxidative phosphorylation deficiency 18, CPOX: Coproporphyrinogen oxidase, CYP450: Cytochrome P450, Cyt b: Cytochrome b, Cyt c1: Cytochrome c1, DFO: Deferoxamine, DMK: dimethyl-α-ketoglutarate, DMSO: Dimethyl sulfoxide, DMT1: Divalent metal transporter 1, dSfxn: Drosophila sideroflexin, ETC: Electron transport chain, Fe^2+^: Iron ferrous, Fe^3+^: Iron ferric, Fe-S: Iron–sulfur, FECH: Ferrochelatase, FeSFA: Fluorescence assay, FIN56: ferroptosis inducing 56, FLVCR1b: Feline leukemia virus subgroup C receptor 1, Fp: Flavoprotein, fpn-1.1: Ferroportin 1.1, Fsf1: Fungal sideroflexin 1, FtMt: Ferritin Mitochondrial, FXN: Frataxin, GDH: Glutamate dehydrogenase, Gpx4: Glutathione peroxidase 4, GTP: Guanosine Triphosphate, HBM: Heme Binding Motif, HEK: Human embryonic kidney, HO-1: Heme oxygenase-1, ISCs: Iron-sulfur clusters, IMM: Inner mitochondrial membrane, IMPC: International Mouse Phenotyping Consortium, IMS: Intermembrane space, IRE: Iron Response Elements, IRP1/2: Iron Related Protein 1 and 2, ISCU: Iron-sulfur cluster assembly enzyme, ISP: Iron-sulfur protein, LC-MS/MS: Liquid chromatography-coupled to tandem mass spectrometry, LPS: Lipopolysaccharide, LYRM7: LYR motif-containing protein 7, Madh5: Mothers against decapentaplegic homolog, MDA: Malondialdehyde, MEF: Mouse Embryonic Fibroblasts, MEL: Mouse erythroleukemia, Mfrn1/2: Mitoferrin 1/2, MnSOD: Manganese superoxide dismutase, NAD(P)+: Nicotinamide adenine dinucleotide phosphate, NADH: Nicotinamide adenine dinucleotide hydrogen, NCO4A: nuclear receptor coactivator 4, NDUFB8: NADH dehydrogenase [ubiquinone] 1 beta subcomplex subunit 8, NDUFS1: NADH dehydrogenase (ubiquinone) Fe-S protein 1, NDUFS7: NADH dehydrogenase (ubiquinone) Fe-S protein 7, NDUFS8: NADH dehydrogenase (ubiquinone) Fe-S protein 8, NDUFV1: NADH dehydrogenase [ubiquinone] flavoprotein 1, NFS1: nitrogen fixation 1 homolog (S. cerevisiae), NMJ: Neuromuscular junctions, NUDFV2: NADH dehydrogenase [ubiquinone] flavoprotein 2, OCM: One-carbon metabolism, OCR: Oxygen Consumption Rates, OXPHOS: Oxidative Phosphorylation, PD: Parkinson’s disease, PGB: Porphobilinogen, PL-PUFA: phospholipid-bound polyunsaturated fatty acids, PPIX: Protoporphirin IX, PPOX: Protoporphyrinogen oxidase, PTGS2: Prostaglandin-Endoperoxide Synthase 2, RC: Respiratory complexes, RCD: Regulated Cell death, RISP: Rieske iron-sulfur protein, ROS: Reactive oxygen species, RSL3: Ras-selective lethality protein 3, SCHAD: Short-Chain 3-Hydroxyacyl-Coenzyme A, SDHA: Succinate dehydrogenase complex, subunit A, SDHB: Succinate dehydrogenase complex, subunit B, SDHC: Succinate dehydrogenase complex, subunit C, Ser: Serine, SFXN: Sideroflexins, SHMT2: Serine Hydroxymethyltransferase 2, SILAC: Stable isotope labeling by amino acids, SIRT4: Sirtuin 4, SLC25A38 Solute carrier Family 25 Member 38, SLC25A39: Solute Carrier Family 25 Member 39, SLC56: Solute carrier family, STEAP3: Six-Transmembrane Epithelial Antigen of Prostate 3, STED: Stimulation Emission Depletion, TCA: Tricarboxylic acid, TCC: Tricarboxylate carrier, TEM-EDX: Transmission electron microscopy linked with energy-dispersive X-ray spectroscopy, TF: Transferrin, TFR1: Transferrin receptor protein 1, TIM22: Translocase of Inner Mitochondrial Membrane 22, TTC19: Tetratricopeptide Repeat Domain 19, UQCC1-3: ubiquinol-cytochrome c reductase complex assembly factor 1, UQCRC2: Cytochrome b-c1 complex subunit 2, UQCRFS1: Cytochrome b-c1 complex subunit Rieske, UROD: Uroporphynogen decarboxylase, UROgenIII: Uroporphyrinogen III, UROS: Uroporphyrinogen Synthase.

## Appendix A

### Appendix A.1. Prediction of Heme Binding Motifs in SFXN1

The web interface HeMoQuest (131.220.139.55/SeqDHBM/) was used for the determination of heme binding motifs in human SFXN1 (Uniprot entry Q96NB2). The default mode released 13 possible heme-coordination sites and the WESA mode, which passes the sequence through a sequence-based solvent accessibility meta-predictor [121,122], gave 4 putative HBMs. These putative HBMs were further located on SFXN1 predicted structure, highlighting 4 sites that may transiently interact with heme (Figure A1). If our predictions are correct, two sites would be located in the matrix and the others would be in the intermembrane space.

### Appendix A.2. Mitochondrial Labile Iron Staining with the Mito-FerroGreen Fluorescent Probe

Prior to the staining, HT1080 cells were seeded in a 6-well plate and transiently transfected with a validated scrambled control siRNA (Control siRNA-A sc-37007, Santa Cruz Biotechnology, INC, Heidelberg, Deustchland)) or a pool of specific siRNA for SFXN1 (sc-91814, Santa Cruz Biotechnology, INC) using Interferin™ transfection reagent (Polyplus-transfection Inc., New York, NY, USA) following manufacturer instructions. Briefly, a mix of siRNA and Interferin™ transfection reagent was prepared and incubated for 10 min at room temperature, and then, added to each well at a final concentration of 10 nM. Cells were incubated at 37 °C under standard culture conditions and amplified. 24 h post-transfection, cells were seeded in µ-Slide 2 Well (Ibidi) and further incubated at 37 °C under standard culture conditions for 24 h. The following day, cells were eventually treated with deferoxamine (DFO) with or without FeCl3 for 1h30 or erastin for 6 h before adding the Mito-FerroGreen probe (Dojindo EU Gmbh, TEBU, Le Perray-en-Yvelines, France). Mito-FerroGreen staining was done according to the manufacturer’s recommandations. Live imaging images were acquired on a Leica TCS SPE confocal microscope with a HC PL APO CS2 63X/1.4 oil immersion objective (CYMAGES imaging facility, UVSQ, Montigny-Le-Bretonneux, France). Image analysis of three independent experiments was done using ImageJ software with a macro developed in the lab.
